# Improving Maximal Strength in the Initial Postoperative Phase After Anterior Cruciate Ligament Reconstruction Surgery: Randomized Controlled Trial of an App-Based Serious Gaming Approach

**DOI:** 10.2196/14282

**Published:** 2020-01-24

**Authors:** Jan-Dierk Clausen, Niclas Nahen, Hauke Horstmann, Florian Lasch, Werner Krutsch, Christian Krettek, Thomas Sanjay Weber-Spickschen

**Affiliations:** 1 Trauma Department Hannover Medical School Hannover Germany; 2 Orthopaedic Surgery Department Hannover Medical School Hannover Germany; 3 Institute of Biometry Hannover Medical School Hannover Germany; 4 Trauma Department University Medical Center Regensburg Regensburg Germany

**Keywords:** serious gaming, knee trainer, games, experimental, exercise therapy, physical and rehabilitation medicine, anterior cruciate ligament reconstruction, knee injuries

## Abstract

**Background:**

Anterior cruciate ligament reconstruction surgery is one of the most common orthopedic procedures. One of the main factors that influence the outcome is regaining strength in the postoperative phase. Because anterior cruciate ligament reconstruction surgeries are often performed in young patients, we combined the concept of prehabilitation with an app-based serious gaming approach to improve maximal strength postoperatively.

**Objective:**

Our objective was to conduct a prospective randomized trial to evaluate whether an app-based active muscle training program (GenuSport Knee Trainer) can improve postoperative strength by starting rehabilitation immediately after primary anterior cruciate ligament reconstruction surgery.

**Methods:**

We designed a pilot study in which we randomly assigned patients receiving primary anterior cruciate ligament reconstruction to either the serious gaming training (intervention) group or a conventional rehabilitation (control) group. Except for the serious gaming-based training, both groups followed the same postoperative treatment protocol. Outcome parameters were absolute and relative change in maximal strength, as well as the International Knee Documentation Committee Subjective Knee evaluation form, Knee Injury and Osteoarthritis Outcome Score, and Lysholm Knee Score.

**Results:**

In total 26 patients agreed to participate (14 patients in the intervention group and 12 patients in the control group, 1 of whom was lost to follow-up). We noted a difference in absolute maximum strength between the exergaming intervention and the control groups. Mean maximum strength preoperatively was 155.1 (SD 79.2) N in the intervention group (n=14) and 157.0 (SD 40.8) N in the control group (n=11). Postoperative mean maximum strength was 212.8 (SD 78.5) N in the intervention group and 154.5 (SD 27.1) N in the control group. Mean absolute change in maximum strength was 57.7 (SD 95.2) N in the intervention group and –4.8 (22.2) N in the control group. The analysis of covariance model with absolute change as the dependent variable and treatment group and baseline maximum strength as covariates showed a relevant difference in relative change between treatment groups (intervention – control) of 59.7 N (95% CI 10.1-109.3; *P*=.02). Similarly to the absolute increase, the relative change in maximum strength was relevantly higher in the exergaming group. The mean relative change in maximum strength was 1.7 (SD 1.17) in the intervention group and 1 (SD 0.13) in the control group. No adverse events or problems were reported during the study period.

**Conclusions:**

Implementation of an app-based active muscle training program in the early postoperative therapy scheme was associated with an improvement in maximal strength. Therefore, we considered the use of GenuSport training after anterior cruciate ligament reconstruction to be a helpful complement to rehabilitation after anterior cruciate ligament reconstruction surgery to improve strength in the early postoperative phase. To our knowledge this was the first study to analyze immediate postoperative serious gaming-based training with the GenuSport device based on strength improvement.

## Introduction

### Background

The rupture of the anterior cruciate ligament (ACL) is one of the most common ligament sports injuries of the knee joint. The number of cruciate ligament operations performed is quite high and has increased continuously over the last years. Because ACL ruptures often occur during sport activities, a relevant number patients are under the age of 40 years [1].

Over the past years, many changes have been achieved, especially in surgical reconstruction techniques. Nonetheless, especially in the last decade, studies have also focused on rehabilitation after ACL reconstruction and on possible strategies to improve outcomes. It is well known that, in addition to several other preoperative and postoperative factors, insufficient rehabilitation leads to a poor outcome in terms of restricted range of motion, strength, and overall knee function, resulting in arthrofibrosis [2-4]. Due to these improvements, the risk of arthrofibrosis has been reduced to around 5% [5].

The tremendous importance of early rehabilitation in all fields of medicine, for example, after neurological disorders (eg, stroke) or surgeries in general, has been understood over the last decades. That is why new concepts have been implemented in the perioperative and postoperative phases. The first concept of enhanced recovery after surgery, consisting mostly of protocols in which patients were guided by physiotherapists, already led to a clinically significant improvement in rehabilitation [6-8]. New concepts have been developed focusing on training, in which patients train at home under the guidance of a physiotherapist (telerehabilitation). This concept is already well accepted in the case of total knee arthroplasty and has shown results comparable with normal outpatient rehabilitation protocols [9,10]. Along with the concept of telerehabilitation, new training devices are being used in rehabilitation, making direct biofeedback-guided training therapy at home possible [11-13]. This biofeedback-guided training has now been combined with the concept of exergaming, in which patients train with devices in conjunction with app-based systems that allow patients to train specific aspects such as range of motion or strength by successfully completing games optimized for the each training process [14,15].

One problem of most available feedback-controlled training systems is that the exercises mainly focus on range of motion, and training is only possible with an acceptable range of motion. Therefore, we previously tested a device in rehabilitation after total knee arthroplasty focusing on feedback-controlled active muscle training uncoupled from range of motion. The GenuSport Knee Trainer improved the outcome after total knee arthroplasty especially in the immediate postoperative phase [16]. Additionally, high compliance rates were noted [16-19].

### Objective

We postulated that training with our device in the initial postoperative phase after ACL reconstruction surgery would improve the overall postoperative outcome, thereby helping the predominantly young patients on their return to normal life and especially to an early return to sports. In comparison with other studies that mainly focused on improved range of motion rather than strength improvement, our device can be used directly after surgery [20]. This allows for a fast-track rehabilitation concept that combines the idea of prehabiliation with serious exergaming strategies.

To our knowledge, no studies have analyzed the effect of muscle strength–based training immediately after ACL surgery. We developed this randomized controlled trial to address this lack.

## Methods

### Study Design

In this prospective randomized controlled trial, we recruited patients awaiting primary ACL reconstruction surgery at a single tertiary health care center between April 2016 and February 2018. The trial is registered; Multimedia Appendix 1 [21]).We obtained ethical approval from the Hanover Medical School ethics committee. Due to different surgical approaches and postoperative treatment protocols, we included patients between 13 and 46 years of age. The main exclusion criteria were additional knee injuries that altered the postoperative treatment protocol (such as meniscal suturing, collateral ligament repair, or regenerative cartilage treatment) and unwillingness to participate in the study. We used computer-based randomization by generating a list of randomized numbers that were provided in sealed envelopes by an independent examiner. The postoperative treatment protocol was identically standardized, apart from the use of the GenuSport Knee Trainer by the intervention group. Pain management was the same for all patients, and none of the patients received a continuous peripheral nerve block. The postoperative physiotherapy protocol included gait training, assisted walking with crutches, active and passive knee mobilization, strength exercises, and stair climbing. In the training intervention group, each patient was additionally provided with a GenuSport Knee Trainer device (prototype plus tablet with software app) with the active knee extension training program for 3 weeks. Otherwise, the postoperative protocol was identical in both the intervention and control groups. Patients were required to train up to 5 times daily with the knee trainer starting on the day of surgery.

### GenuSport Knee Trainer

**Figure 1:**
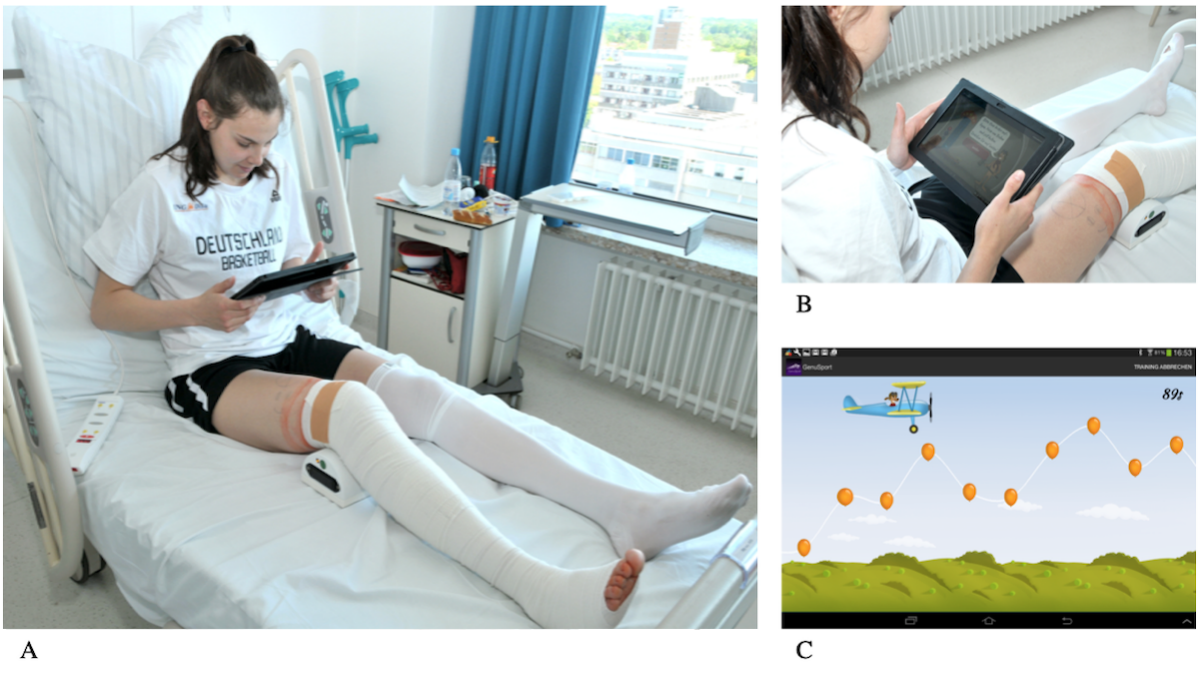
The GenuSport knee trainer. (A) The device is correctly placed under the patient’s knee. (B) The patient holds the tablet while in an upright position. (C) The mobile app shows the game (lasting 100 s) in which the patient controls the flight course of an airplane by applying various forces. The aim is to destroy the balloons with the propeller and to avoid the dark clouds.

We previously described the Knee Trainer device [16-19]. It consists of a strength-monitoring unit with 3 integrated sensors that is placed in the popliteal area and a tablet with the app, which transfers the raised force into the game modus (Figure 1). Each training session in our study takes around 5 minutes and is performed autonomously by patients in their bed with 45° upper-body elevation while the patient holds the tablet in both hands. By simply pushing the knee downward onto the measuring unit, the patient can apply force. It is of tremendous importance that during the entire training session, the leg is not rotated, the hip is not lifted, and the heel is in contact with the mattress. The training app has 2 modes: 1 for the training itself and 1 for analysis. The training mode includes 2 different games: 1 for maximal strength, in which the patient has to apply maximal force for 5 seconds, and 1 longer game lasting 100 seconds, in which the patient controls the flight course of an airplane by applying different forces. The aim is to destroy the balloons with the propeller and to avoid the dark clouds. Direct feedback is provided after each game on a summary screen presenting the results [17].

### Blinding

Due to the nature of this study, blinding of patients was not possible.

### Intervention

Surgeries were performed by the same senior physician. In all patients, hamstring grafts were used. The fixation technique included femoral and tibial screw fixation (Storz Medical AG, Tägerwilen, Switzerland), as well as extracortical fixation (Storz Medical).

### Outcomes

We analyzed all patients before and 6 weeks after surgery. We measured function using the International Knee Documentation Committee Subjective Knee (IKDC) evaluation form, Lysholm Knee Score, Tegner activity scale, Knee Injury and Osteoarthritis Outcome Score (KOOS), the maximum strength, and a visual analog scale. Additionally, we measured the femoral diameter 10 cm and 20 cm above the joint line.

We analyzed change in maximum strength as both the absolute difference between the week-6 and presurgery values (6 weeks – presurgery) and as the relative change (6 weeks / presurgery). We compared the change in maximum strength on both scales between the intervention and control groups using an analysis of covariance (ANCOVA) model with change in maximum strength as the dependent variable and presurgical maximum strength and treatment group as the independent variables.

### Statistical Evaluation

We performed statistical analysis using IBM SPSS Statistics version 25 (IBM Corporation).

## Results

### Participants

**Figure 2:**
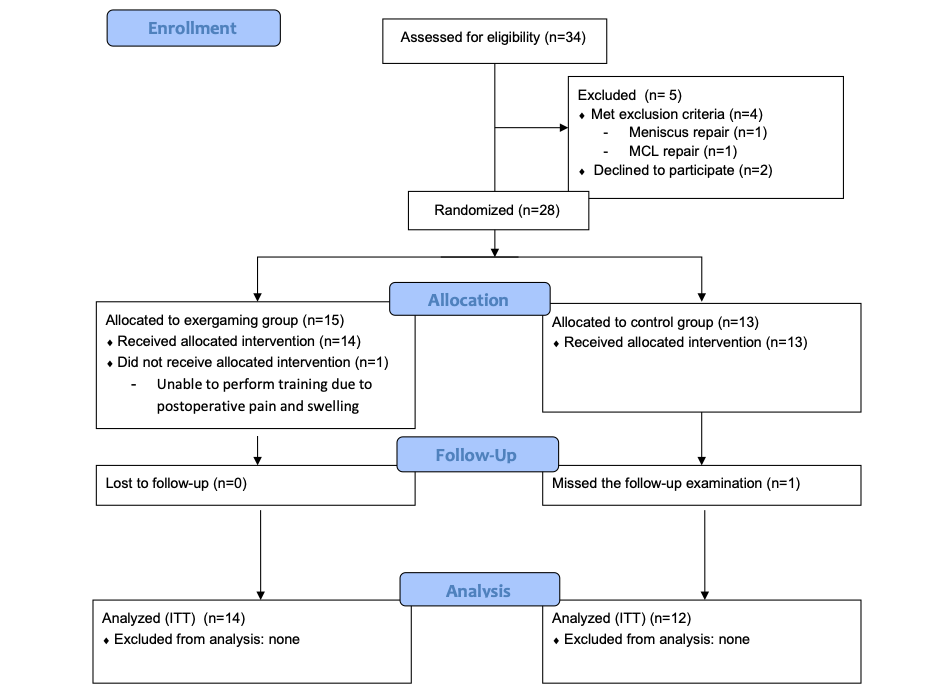
Consolidated Standards of Reporting Trials (CONSORT) study flow diagram. ITT: intention to treat; MCL: medial collateral ligament.

In total 26 patients agreed to participate (14 patients in the exergaming group and 12 patients in the control group). One patient of the control group was lost to follow-up; hence, we analyzed 25 patients in total (14 intervention patients vs 11 control group patients) (Figure 2).

### Baseline Characteristics

Table 1 lists the baseline characteristics of the included patients regarding age, sex, body mass index and preoperative scores and strength. The 2 groups did not differ in terms of these criteria.

### Compliance in the Knee Trainer Group

We saw high compliance rates within the intervention group. None of the patients needed more than 1 instruction to use the knee trainer. Of the 14 patients in this group, 11 (79%) trained with the device regularly (around 17 times a week), whereas 3 patients (20%) did not comply with the training frequency of at least 7 times a week.

### Clinical Outcomes

**Figure 3:**
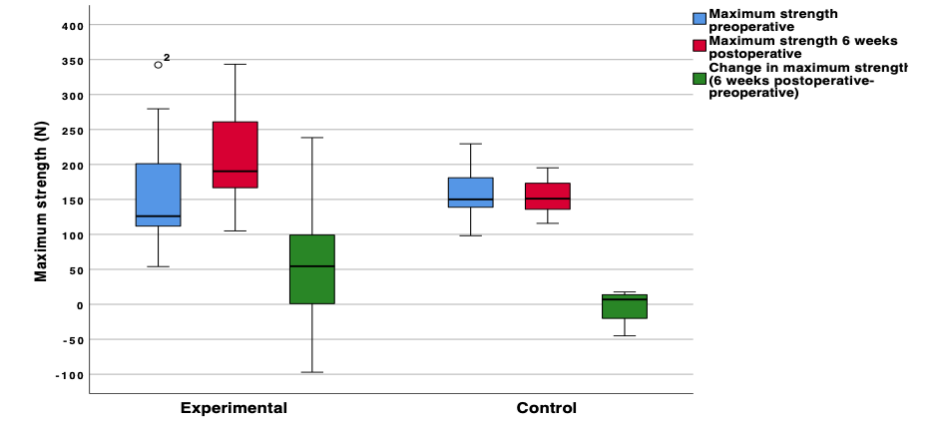
Box and whisker plot of the experimental and control groups comparing absolute change in maximum strength 6 weeks after surgery versus before surgery (6 weeks – preoperative), depicting the 25th to 75th percentile in the box with the mean indicated by the bar in the box. The whiskers indicate the 10th and 90th percentiles. One outlier in the experimental group is depicted by the circle.

Analysis of the data showed a relevant difference in the absolute increase of maximum strength between the exergaming group (n=14) and the control group (n=11). Postoperative mean maximum strength was 212.8 (SD 78.5) N in the intervention group (n=14) and 154.5 (SD 27.1) N in the control group (n=11). Mean absolute change in maximum strength was 57.7 (SD 95.2) N in the intervention group and –4.8 (SD 22.2) N in the control group. The ANCOVA model with absolute change as the dependent variable and treatment group and baseline maximum strength as covariates showed a relevant difference in absolute change between treatment groups (experimental – control) of 59.7 N (95% CI 10.1-109.3; *P*=.02) (Figure 3).

**Table 1:** Baseline characteristics and outcomes of the study groups.

Characteristics		Intervention group (n=14)	Control group (n=12)	Total (n=26)
Sex (women), n (%)		8 (57)	6 (50)	14 (54)
Body mass index (kg/m^2^), mean (SD)		24.6 (5.74)	25.45 (4.02)	25.45 (4.02)
Age (years), mean (SD)		24.86 (9.71)	25.58 (6.4)	25.19 (8.2)
Tegner activity scale score, mean (SD)		7 (2.25)	6.33 (1.61)	6.69 (1.98)
Time between operation and final examination (days), mean (SD)		46.50 (8.58)	44.92 (5.53)	45.77 (7.24)
**GenuSport Knee Trainer maximum strength (kg), mean (SD)**				
	Operated leg	15.81 (8.06)	16 (4.16)	15.90 (6.44)
	Nonoperated leg	17.73 (6.59)	17.62 (3.91)	17.68 (5.42)
**IKDC^a^ 2000 subjective, mean (SD)**		50.16 (16.10)	61.11 (14.34)	55.22 (16.00)
	Symptoms/stiffness	53.32 (22.91)	65.77 (18.56)	59.19 (20.08)
	Pain	63.52 (24.03)	70.37 (19.55)	66.68 (21.92)
	Function/daily living	71.64 (23.63)	83.16 (14.78)	76.71 (20.68)
	Function/sports	31.16 (26.18)	48.13 (25.74)	38.99 (26.87)
	Quality of life	22.32 (16.02)	31.94 (17.16)	26.76 (16.94)
Lysholm activity scale score, mean (SD)		55.79 (21.51)	63.17 (18.37)	59.19 (20.08)
Single assessment numeric evaluation, mean (SD)		49.93 (28.06)	61.25 (18.23)	55.15 (24.26)
Visual analog scale score, mean (SD)		2.61 (2.34)	2.53 (1.74)	2.57 (2.04)

^a^IKDC: International Knee Documentation Committee Subjective Knee evaluation form, 2000 revision.

**Figure 4:**
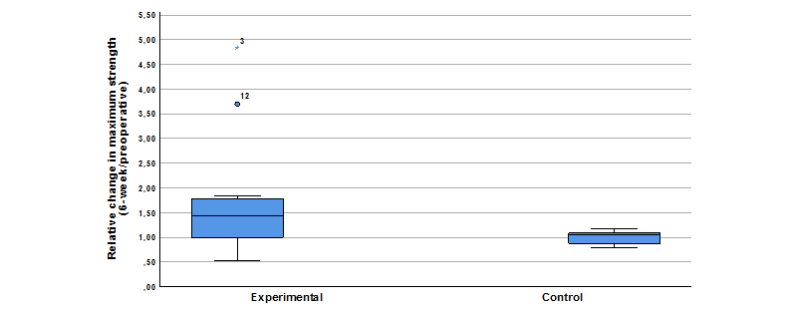
Box and whisker plot of the experimental and control groups comparing relative change in maximum strength 6 weeks after surgery versus before surgery (6 weeks / preoperative), depicting the 25th to 75th percentile in the box with the mean indicated by the bar in the box. The whiskers indicate the 10th and 90th percentiles. Two outliers in the experimental group are depicted by the circle and the star.

Relative change in maximum strength was higher in the exergaming group. Mean relative change in maximum strength was 1.7 (SD 1.17) in the intervention group and 1 (SD 0.13) in the control group. The ANCOVA model with relative change as the dependent variable and treatment group and baseline maximum strength as covariates showed a relevant difference in relative change between treatment groups (experimental – control) of 0.67 (95% CI 0.054-1.299; *P*=.03) (Figure 4).

A subgroup analysis of patients who trained more than 2 times a day showed no difference in absolute and relative changes in maximum strength, but we saw a clear trend to higher strength levels in the group of patients that trained more than 2 times a day.

Changes in clinical scores (eg, IKDC, KOOS, Tegner activity scale, Lysholm Knee Score, and visual analog scale) were not different between the 2 study groups (Table 1).

### Adverse Events

Neither group reported adverse events during the study period. In particular, there were no readmissions to the hospital, reoperation for any reason, infections, or falls.

## Discussion

### Principal Findings

Following the progress made in information and communication technology, new technology-based rehabilitation strategies have been incorporated into various medical fields. In this context, new protocols for rehabilitation have been developed. These serious games for functional rehabilitation are considered to improve the whole rehabilitation process. Notably, in most published studies, patient compliance has been very high. This indicates a high motivation for training with exergaming devices. These results for serious games have been reported in all fields of medicine (eg, psychiatry, internal medicine, neurology, and musculoskeletal medicine) [22-25]. Although our patient population was quite small, the high compliance rate we observed is comparable with previous studies.

All of the patients allocated to the exergaming group completed the entire follow-up protocol. In the exergaming intervention group, about 80% showed good compliance, meaning they trained at least once a day, and none of these patients needed face-to-face contact during the 3-week training period. This indicates that the device design is user-friendly and that people from different age groups can use the device autonomously after adequate initial instruction. Ideally, instruction would start before surgery. We think that there is also potential for using the device in the preoperative phase to improve preoperative strength, which is known to positively influence the postoperative outcome [3,26,27].

We saw a slight trend in the frequency of daily training decreasing over the 3 weeks. We think that this might have been because the gameplay of the 2 game modes that were provided did not develop over time in difficulty or the main game principle. To achieve the goal of implementing serious gaming into the rehabilitation of musculoskeletal disorders, existing systems must be evaluated and further developed [22]. To this end, we are developing a 3-level system with the difficulty increasing at every level. We also think that the patient’s fitness needs to be evaluated preoperatively and that the game difficulty would be adapted to the patient’s fitness level. We are also trying to implement different surroundings, such as desert, space, and ocean.

The most important problem in feedback training strategies is that nearly all of them require an adequate range of motion. Therefore, therapy cannot start immediately after surgery because the patient is not able to properly move his or her legs. We designed our study to assess whether it is possible to positively influence the immediate postoperative phase by following a novel exergaming-based training protocol based on strength training rather than on range of motion as in other protocols. Other recently published studies of feedback-controlled training after total knee arthroplasty started their programs not before day 7 [13]. Results of feedback-controlled range-of-motion training were very promising and showed significant improvements in the range-of-motion outcome [20]. In comparison with range-of-motion protocols, the most important takeaway from our study is the relevant improvement in the maximum strength relative to that obtained with the conventional rehabilitation protocol. Regaining strength is an important parameter in the functional outcome [28]. To our knowledge, the protocol we analyzed is unique in that it is possible to train immediately after surgery. We therefore think that our protocol can help to improve the overall outcome in addition to range-of-motion–based strategies. These aspects need to be tested in further studies, and the device should also be combined with training devices focusing on range of motion.

Because serious gaming has already been incorporated into other fields of medicine with good results, musculoskeletal surgeons should also implement these promising strategies both before surgery and into rehabilitation after common procedures, as well as in conservative orthopedics. The good results in different age groups suggest that serious gaming is applicable in nearly all periods of life [29-31].

### Limitations

The main limitations of our study were a comparatively small patient population and a short follow-up period of 6 weeks. This is due to the fact that our approach to the immediate postoperative strength-based training has not been implemented in postoperative protocols. Hence, we conducted this pilot study to evaluate the effect of exergaming-based training on the early postoperative phase as a basis for further studies.

### Conclusion

To our knowledge, this was the first study that successfully implemented a serious gaming-based strength training device into early rehabilitation after ACL surgery. The results are promising; hence, we think that this technology has high potential in this area of rehabilitation, as well as in prehabilitation of musculoskeletal disorders. Further studies are needed to test these concepts and results. If the results of these further studies are as promising as the results of our study, we think that this might be a big step toward implementing such training strategies into routine clinical protocols, especially since the positive aspects of serious gaming are seen in all age cohorts.

## Appendix

Multimedia Appendix 1 CONSORT-EHEALTH checklist V1.6.1.

## Abbreviations

ACL: anterior cruciate ligament
ANCOVA: analysis of covariance
IKDC: International Knee Documentation Committee Subjective Knee
KOOS: Knee Injury and Osteoarthritis Outcome Score

## References

[1] Frobell RB, Roos EM, Roos HP, Ranstam J, Lohmander LS (2010). A randomized trial of treatment for acute anterior cruciate ligament tears. N Engl J Med.

[2] Frobell RB, Roos HP, Roos EM, Roemer FW, Ranstam J, Lohmander LS (2013). Treatment for acute anterior cruciate ligament tear: five year outcome of randomised trial. BMJ.

[3] Grindem H, Granan LP, Risberg MA, Engebretsen L, Snyder-Mackler L, Eitzen I (2015). How does a combined preoperative and postoperative rehabilitation programme influence the outcome of ACL reconstruction 2 years after surgery? A comparison between patients in the Delaware-Oslo ACL Cohort and the Norwegian National Knee Ligament Registry. Br J Sports Med.

[4] Mayr HO, Weig TG, Plitz W (2004). Arthrofibrosis following ACL reconstruction--reasons and outcome. Arch Orthop Trauma Surg.

[5] Christensen JE, Miller MD (2018). Knee anterior cruciate ligament injuries: common problems and solutions. Clin Sports Med.

[6] Ljungqvist O (2014). ERAS--enhanced recovery after surgery: moving evidence-based perioperative care to practice. JPEN J Parenter Enteral Nutr.

[7] Ljungqvist O, Scott M, Fearon KC (2017). Enhanced recovery after surgery: a review. JAMA Surg.

[8] Ljungqvist O, Young-Fadok T, Demartines N (2017). The history of enhanced recovery after surgery and the ERAS Society. J Laparoendosc Adv Surg Tech A.

[9] Russell TG, Buttrum P, Wootton R, Jull GA (2011). Internet-based outpatient telerehabilitation for patients following total knee arthroplasty: a randomized controlled trial. J Bone Joint Surg Am.

[10] Moffet H, Tousignant M, Nadeau S, Mérette C, Boissy P, Corriveau H, Marquis F, Cabana F, Belzile ÉL, Ranger P, Dimentberg R (2017). Patient satisfaction with in-home telerehabilitation after total knee arthroplasty: results from a randomized controlled trial. Telemed J E Health.

[11] Pfeufer D, Gililland J, Böcker W, Kammerlander C, Anderson M, Krähenbühl N, Pelt C (2019). Training with biofeedback devices improves clinical outcome compared to usual care in patients with unilateral TKA: a systematic review. Knee Surg Sports Traumatol Arthrosc.

[12] Bahadori S, Wainwright TW, Ahmed OH (2018). Smartphone apps for total hip replacement and total knee replacement surgery patients: a systematic review. Disabil Rehabil.

[13] Correia FD, Nogueira A, Magalhães I, Guimarães J, Moreira M, Barradas I, Molinos M, Teixeira L, Tulha J, Seabra R, Lains J, Bento V (2019). Medium-term outcomes of digital versus conventional home-based rehabilitation after total knee arthroplasty: prospective, parallel-group feasibility study. JMIR Rehabil Assist Technol.

[14] Sims J, Cosby N, Saliba EN, Hertel J, Saliba SA (2013). Exergaming and static postural control in individuals with a history of lower limb injury. J Athl Train.

[15] Siegel SR, Haddock B, Dubois AM, Wilkin LD (2009). Active video/arcade games (exergaming) and energy expenditure in college students. Int J Exerc Sci.

[16] Horstmann H, Krost E, Welke B, Kerling A, Hanke A, Jakubowitz E, Weber-Spickschen TS (2018). The determination of the validity of an application-based knee-training device. Assist Technol.

[17] Hardt S, Schulz MRG, Pfitzner T, Wassilew G, Horstmann H, Liodakis E, Weber-Spickschen TS (2018). Improved early outcome after TKA through an app-based active muscle training programme-a randomized-controlled trial. Knee Surg Sports Traumatol Arthrosc.

[18] Horstmann H, Clausen JD, Krettek C, Weber-Spickschen TS (2017). [Evidence-based therapy for tendinopathy of the knee joint : which forms of therapy are scientifically proven?]. Unfallchirurg.

[19] Horstmann H, Colcuc C, Lobenhoffer P, Krettek C, Weber-Spickschen TS (2017). Evaluation of the acceptability of a sphygmomanometer device in knee extension training following surgical procedures of the knee. Int J Orthop Trauma Nurs.

[20] Cordeiro d'Ornellas M, Santos Machado A, de Moraes JP, Cervi Prado AL (2017). A serious game for anterior cruciate ligament rehabilitation: software development aspects and game engagement assessment. Stud Health Technol Inform.

[21] Eysenbach G, CONSORT-EHEALTH Group (2011). CONSORT-EHEALTH: improving and standardizing evaluation reports of Web-based and mobile health interventions. J Med Internet Res.

[22] Idriss M, Tannous H, Istrate D, Perrochon A, Salle JY, Ho Ba Tho MC, Dao TT (2017). Rehabilitation-oriented serious game development and evaluation guidelines for musculoskeletal disorders. JMIR Serious Games.

[23] Chen P, Wei S, Hsieh W, Cheen J, Chen L, Kao C (2012). Lower limb power rehabilitation (LLPR) using interactive video game for improvement of balance function in older people. Arch Gerontol Geriatr.

[24] Amado I, Brénugat-Herné L, Orriols E, Desombre C, Dos Santos M, Prost Z, Krebs M, Piolino P (2016). A serious game to improve cognitive functions in schizophrenia: a pilot study. Front Psychiatry.

[25] Tannous H, Istrate D, Benlarbi-Delai A, Sarrazin J, Idriss M, Ho Ba Tho MC, Tien Tuan Dao (2016). Exploring various orientation measurement approaches applied to a serious game system for functional rehabilitation. Conf Proc IEEE Eng Med Biol Soc.

[26] Grindem H, Risberg MA, Eitzen I (2015). Two factors that may underpin outstanding outcomes after ACL rehabilitation. Br J Sports Med.

[27] Heijne A, Ang BO, Werner S (2009). Predictive factors for 12-month outcome after anterior cruciate ligament reconstruction. Scand J Med Sci Sports.

[28] Csapo R, Hoser C, Gföller P, Raschner C, Fink C (2018). Fitness, knee function and competition performance in professional alpine skiers after ACL injury. J Sci Med Sport.

[29] Labruyère R, Gerber CN, Birrer-Brütsch K, Meyer-Heim A, van Hedel HJA (2013). Requirements for and impact of a serious game for neuro-pediatric robot-assisted gait training. Res Dev Disabil.

[30] Bower KJ, Louie J, Landesrocha Y, Seedy P, Gorelik A, Bernhardt J (2015). Clinical feasibility of interactive motion-controlled games for stroke rehabilitation. J Neuroeng Rehabil.

[31] Manera V, Petit P, Derreumaux A, Orvieto I, Romagnoli M, Lyttle G, David R, Robert PH (2015). 'Kitchen and cooking,' a serious game for mild cognitive impairment and Alzheimer's disease: a pilot study. Front Aging Neurosci.

